# The Effect of Supplementation with Omega-3 Polyunsaturated Fatty Acids on Markers of Oxidative Stress in Elderly Exposed to PM_2.5_

**DOI:** 10.1289/ehp.10578

**Published:** 2008-05-16

**Authors:** Isabelle Romieu, Raquel Garcia-Esteban, Jordi Sunyer, Camilo Rios, Mireya Alcaraz-Zubeldia, Silvia Ruiz Velasco, Fernando Holguin

**Affiliations:** 1 Instituto Nacional de Salud Pública, Cuernavaca, Morelos, México; 2 Instituto Municipal de Investigaciones Médicas and Centre de Recerca en Epidemiologia Ambiental, Barcelona, Spain; 3 Instituto Nacional de Neurología, México DF, México; 4 Instituto de Investigaciones en Matemáticas Aplicadas y en Sistemas, Universidad Nacional Autónoma de México, México DF, México; 5 Emory University School of Medicine, Atlanta, Georgia, USA

**Keywords:** biological markers, omega-3 polyunsaturated fatty acids (PUFA), oxidative stress, PM_2.5_

## Abstract

**Background:**

The mechanisms of particulate matter (PM)-induced health effects are believed to involve inflammation and oxidative stress. Increased intake of omega-3 polyunsaturated fatty acids (n-3 PUFA) appears to have anti-inflammatory effects.

**Objective:**

As part of a trial to evaluate whether n-3 PUFA supplementation could protect against the cardiac alterations linked to PM exposure, we measured biomarkers of response to oxidative stimuli [copper/zinc (Cu/Zn) superoxide dismutase (SOD) activity, lipoperoxidation (LPO) products, and reduced glutathione (GSH)] and evaluated the impact of supplementation on plasma levels.

**Methods:**

We recruited residents from a nursing home in Mexico City chronically exposed to PM ≤2.5 μm in aerodynamic diameter (PM_2.5_) and followed them from 26 September 2001 to 10 April 2002. We randomly assigned subjects in a double-blind fashion to receive either fish oil (n-3 PUFA) or soy oil. We measured PM_2.5_ levels indoors at the nursing home, and measured Cu/Zn SOD activity, LPO products, and GSH at different times during presupplementation and supplementation phases.

**Results:**

Supplementation with either fish or soy oil was related to an increase of Cu/Zn SOD activity and an increase in GSH plasma levels, whereas exposure to indoor PM_2.5_ levels was related to a decrease in Cu/Zn SOD activity and GSH plasma levels.

**Conclusion:**

Supplementation with n-3 PUFA appeared to modulate the adverse effects of PM_2.5_ on these biomarkers, particularly in the fish oil group. Supplementation with n-3 PUFA could modulate oxidative response to PM_2.5_ exposure.

Environmental exposure to particulate matter (PM) has been associated with increased cardiovascular mortality in the elderly (Aga et al. 2003; Devlin et al. 2003; Samet et al. 2000) and reductions in heart rate variability (HRV), a measure of cardiac autonomic regulation (Gold et al. 2000; Holguin et al. 2003; Park et al. 2005). Although not well understood, the mechanisms of PM-induced health effects are believed to involve inflammation and oxidative stress initiated by the formation of reactive oxygen species (ROS) within affected cells (Cho et al. 2005; Gonzalez-Flecha 2004). *In vitro* studies have shown that inhaled PM causes expression of nuclear factor kappa-B (NF-κB)-related genes and oxidant-dependent NF-κB activation (Jimenez et al. 2000; Shukla et al. 2000). To defend against the oxidative damage, cells use up their stores of a key antioxidant, glutathione; glutathione depletion can induce a state of cellular stress and the activation of additional intracellular signaling cascades that regulate the expression of cytokine and chemokine genes and widespread proinflammatory effects remote from the site of damage (Saxon and Diaz-Sanchez 2005). Data from PM samples of 20 European cities have shown that PM with an aerodynamic diameter ≤2.5 μm (PM_2.5_) has strong redox activity and is able to deplete artificial respiratory lining fluid of reduced glutathione (GSH) and ascorbate (Künzli et al. 2006). PM, depending on its toxicity, seems also to inhibit protective enzymes involved in oxidative stress responses [copper/zinc (Cu/Zn) superoxide dismutase (SOD), manganese SOD, glutathione peroxidase (GSH-Px), and glutathione reductase] (Hatzis et al. 2006). Alteration of autonomic function related to PM_2.5_ exposure appears to be partly associated with oxidative stress (Brook et al. 2003; Chahine et al. 2007).

Increased intake of omega-3 polyunsaturated fatty acids (n-3 PUFA) has been shown to decrease the risk of cardiovascular events (GISSI Prevenzione Investigators 1999; Kris-Etherton et al. 2002; Leaf et al. 2003). The protective effect of n-3 PUFA seems to be linked in part to its cardiac antiarrhythmic properties (Christensen et al. 2001; Holguin et al. 2005; Kris-Etherton et al. 2002; Leaf 2001) and its anti-inflammatory effects (Calder 2006). Long-chain n-3 PUFA appears to act both directly (by replacing arachidonic acid as an eicosanoid substrate and inhibiting arachidonic acid metabolism) and indirectly by altering the expression of inflammatory genes through effects on transcription factor activation (Calder 2006). Fish oil has been shown to modulate endothelial activation possibly by reducing ROS and therefore leading to the subsequent inactivation of the NF-κB system of gene transcription. This role of oxygen scavenging would lead to the prevention of O_2_-generating hydrogen peroxide (H_2_O_2_) and thus prevent cell activation (De Caterina et al. 2000).

We have previously shown that exposure to PM_2.5_ is related to a decrease in HRV in elderly residents of a nursing home in Mexico City (Holguin et al. 2003) and that fish oil supplementation could increase HRV (Holguin et al. 2005) and modulate the adverse effects of PM_2.5_ on HRV (Romieu et al. 2005). We hypothesized that one of the mechanisms by which fish oil and, to a lesser extent, soy oil supplementation would modu late the adverse effects of PM_2.5_ on HRV would be by acting on the oxidative stress response, reducing the generation of ROS, modulating the use of GSH as part of the oxidative stress response, and increasing the activity of enzymes involved in response to oxidative stimuli. We tested this hypothesis in a randomized trial of fish oil versus soy oil supplementation to prevent reductions in cardiac autonomic function associated with PM exposure. During the trial, we measured Cu/Zn SOD activity, GSH, and LPO levels in plasma to evaluate the impact of fish oil and soy oil supplementation and exposure to PM_2.5_ on these biomarkers.

## Materials and Methods

### Study population and design

We recruited residents from a nursing home in Mexico City who were chronically exposed to PM_2.5_ and followed them from 26 September 2001 to 10 April 2002. We randomly assigned subjects in a double-blind fashion to receive either fish oil (n-3 PUFA) or soy oil. We conducted the study in two phases: a presupplementation phase of 3 months (27 September to 17 December 2001) and a supplementation phase of 4 months (9 January to 5 April 2002) (Holguin et al. 2005). Before starting supplementation, participants provided a blood sample. During the supplementation phase, we asked subjects to provide three blood samples at 1 month, 3 months, and at the end of the study; seven participants provided only two samples during the supplementation phase. In addition, we measured indoor and ambient PM_2.5_ levels in the nursing home, and each participant kept a diary of daily activities.

**Figure 1:**
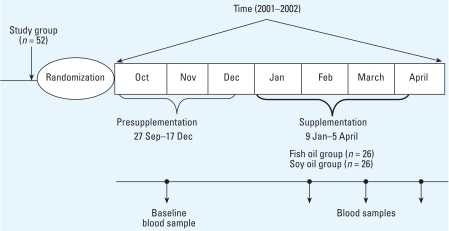
Study design.

The eligibility criteria were age > 60 years, absence of cardiac arrhythmias, no cardiac pacemaker, no allergies to n-3 PUFA or fish, no treatment with oral anticoagulants, no history of bleeding diathesis, and ability to undergo HRV measurements in the supine position. Among 63 residents invited to the study, 52 agreed to participate and provided written informed consent for both randomization and supplementation (for study design, see Figure 1). At baseline, all participants answered to a general purpose questionnaire and a validated food frequency questionnaire administered by a nutritionist (Hernandez-Avila et al. 1998; Romieu et al. 1999). We extracted past medical histories from the medical files. The trial protocol was approved by the Institutional Research Board and Ethical Committee of the National Institute of Public Health in Mexico.

### Randomization to n-3 fish oil or soy oil

We randomly assigned participants to two groups at baseline using a random-number table. Compliance was determined by directly observed supplement intake. Patients in the fish oil group received 2 g/day in divided doses. Each capsule contained 83.2% n-3 PUFA [52.4% docosahexanoic acid (C22:6 n-3 DHA), 25.0% eicosopentanoic acid (C20:5 n-3 EPA) and 5.8% docosapentaenoic acid (c22:5n-3 DPA)]. The soy oil capsules were identical, and each capsule contained 6.78% α-linolenic acid (ALA), a plant n-3 PUFA (C18:3 n-3), 16.3% saturated fat, 52.7% linoleic acid (C18:2 n-6), and 22.5% oleic acid (C18:1 n-9). Neither the participants nor the study personal were aware of the randomization group.

### Pollutants and temperature measurements

We determined daily 24-hr measurements of PM_2.5_ by gravimetric analysis using Mini-Vol portable air samplers (version 4.2; Air Metrics, Eugene, OR, USA), with 47 mm Teflon filters (Pall Gelman Laboratory, Ann Arbor, MI, USA) and flows set at 4 L/min, located within the living room of the nursing home during the follow-up period. Filter gravimetric analysis was performed at the air laboratory of the National Center for Environmental Research and Training in Mexico City under controlled climatic and temperature conditions. Filter weights were obtained by a micrometric scale (Cahn C-35; Thermo Electron Corp., Round Rock, TX, USA) under laminar flow.

We obtained ambient levels of ozone, nitrogen dioxide, sulfur dioxide, and carbon monoxide as well as climatic variables from an automated monitoring station located 3 km upwind from the study site (in Tacuba, to the northeast). Given the low concentrations observed for SO_2_, NO_2_, and CO, we excluded these pollutants from further analysis.

### Laboratory analysis

We immediately centrifuged blood samples, protected plasma aliquots with aluminum foil, and stored them at −70°C until analysis. Laboratory measurements were conducted in duplicate at the laboratory of the National Institute of Neurology in Mexico City. We used the mean of the duplicate measurements as the biomarker values for each individual at each sampling time.

### Cu/Zn SOD activity

We assayed SOD activity by the xanthine/xanthine oxidase method as modified by Alcaraz-Zubeldia et al. (2001) from the method described by Schwartz et al. (1998). We diluted plasma samples in a buffer consisting of 20 mM sodium bicarbonate and 0.02% Triton X-100 (pH 10.2), centrifuged them at 4,000 × *g* for 10 min, and collected the supernatants. We then added 50 μL of the clarified supernatant to 950 μL of the reaction mixture consisting of 10 μM sodium azide, 100 μM xanthine, 10 μM reduced cytochrome c, and 1 mM EDTA in 20 mM sodium bicarbonate, 0.02% Triton X-100 (pH 10.2). We initiated the assay by adding xanthine oxidase and monitored it by measuring the change in absorbance at 560 nm in a Lambda-20 ultraviolet/visible spectrophotometer (Perkin Elmer, Waltham, MA, USA). We carried out the analysis of samples in duplicate and calculated the participation of each SOD type as total activity minus the activity inhibited by the addition of 5 mM sodium cyanide, because cyanide selectively inhibits the Cu/Zn SOD isoform. The results are expressed as international units per milliliter. Measurements of Cu/Zn SOD were available for 20 subjects assigned to the soy oil group and 24 subjects assigned to the fish oil group. The coefficient of variation (mean) of duplicate measurements was 1.6%.

### LPO in plasma

We monitored lipid fluorescence product formation as an index of LPO in plasma samples using the method described by Triggs and Willmore (1984). We separated 500 μL of each plasma sample into a glass tube, covered from light, and added 4 mL of a chloroform-methanol 2:1 mixture. We measured fluorescence in a Perkin Elmer LS50B luminescence spectrophotometer at 370 nm excitation and 430 nm emission. We adjusted sensitivity of the spectrophotometer to 140 fluorescence units with a 0.1-mg/L quinine standard, prepared in 0.05 M aqueous sulfuric acid solution, before the measurement of the samples. We used pooled plasma samples obtained from healthy controls as an internal quality control. We obtained reference values for pooled plasma samples from repeated analyses to obtain quality control charts. Each time we carried out the measurements, we determined the pooled samples in the same sample batch to assess the reproducibility of the measurements. Results are expressed as fluorescence units (FU) per milliliter. We ran all samples in duplicate. Measurements of LPO products were available for 21 subjects assigned to the soy oil group and 21 subjects assigned to the fish oil group. The coefficient of variation (mean) of the duplicate measurements was 10.9%.

### GSH levels in plasma

We assayed GSH using the method described by Hu (1994). We diluted plasma samples 1:1 with a 10% (wt/vol) trichloroacetic solution and placed tubes on ice for 10 min. We measured fluorescence in a Perkin Elmer LS50B luminescence spectrophotometer at 350 nm excitation and 420 nm emission. For blank sample analysis, we substituted plasma samples with deionized water and treated these as regular samples. We performed internal quality control for GSH measurements as described for the lipid fluorescence product assay. We constructed calibration curves for glutathione and obtained the concentrations by interpolation in the standard curve. We express our results as micromolar concentrations of GSH. Measurements of GSH were available for 20 subjects assigned to the soy oil group and 23 subjects assigned to the fish oil group. The coefficient of variation (mean) of the duplicate measurements was 1.8%.

### Statistical analysis

We compared the mean Cu/Zn SOD activity, LPO products, and GSH levels in plasma at baseline between the soy oil and the fish oil groups using a *t*-test. To normalize the distribution, we square-root–transformed the measure of LPO and log-transformed GSH. We determined the effect of supplementation by soil oil and fish oil and of PM_2.5_ exposure on Cu/Zn SOD activity, LPO products, and GSH levels in plasma using linear mixed models (Xu and Hedeker 2001) treating patient as random effect. Linear mixed models account for repeated measurements within the same individual and allow for variation of effect among individuals. We assigned the same-day indoor PM_2.5_ level measured (24-hr average) in the nursing home area as PM_2.5_ exposure for subjects tested on a given day (12 subjects/day). We adjusted regression models only for time, because characteristics of the general population were equally distributed between groups and did not confound the association. We also tested for interaction between PM_2.5_ levels and supplementation phase for both the soy and fish oil supplementation groups.

We assessed the possible nonlinearity of PM_2.5_ using generalized additive mixed models (GAMM) with p-splines (Greven et al. 2006; Hastie and Tibshirani 1990), modeling the dose–response relationship between bio-markers of response to oxidative stimuli and pollutant as a smooth function. Because we observed nonlinear associations between PM_2.5_ and these biomarkers in the fish oil group, we included a quadratic term of PM_2.5_ in our mixed linear model. We assigned the same-day indoor PM_2.5_ level measured in the nursing home area as PM_2.5_ exposure for subjects tested on a given day (12 subjects per day). We used the chi-square or Fisher’s exact test for discrete variables and frequencies, and considered a *p*-value of < 0.05 significant. We conducted statistical analyses using Stata 8.2 (StataCorp., College Station, TX, USA) and GAMM using a SAS macro (version 9.1; SAS Institute Inc., Cary, NC, USA).

## Results

### Study sample

Table 1 presents the general characteristics of participants according to their assignment to either the fish oil or soy oil supplementation groups. Most of the general characteristics were similar between groups. Ischemic cardiac disease was more frequent in the fish oil group (*n* = 0 vs. *n* = 4), and body mass index (BMI) was slightly lower in the fish oil group (27.6 vs. 24.6). Only three participants in the fish oil group and three in the soy oil group reported smoking outside of the residential home (Table 1). At baseline, participants from both the fish oil and soy oil groups reported low dietary intakes of foods rich in n-3 PUFA.

### Environmental exposure data

Participants in both groups spent on average 93% of their time indoors. The mean daily PM_2.5_ ambient level in the living room of the residence was 38.7 μg/m^3^ (14.7 SD), the median was 35.11 μg/m^3^, and the 25th and 75th percentiles were 30.62 μg/m^3^ and 41.10 μg/m^3^, respectively (range, 14.8–70.9 μg/m^3^). Indoor and outdoor PM_2.5_ measurements were highly correlated (*r* = 0.95). We obtained O_3_, PM_10_ outdoor levels, and climatic data from the closest monitoring station. During the study phase, the O_3_ 1-hr maximum ranged from 11.6 to 57.5 ppb, and the PM_10_ 24-hr average ranged from 39.5 to 109.9 μg/m^3^.

### Fatty acids in erythrocytes

In this study, we were not able to measure erythrocyte n-3 PUFA levels. However, a study conducted in a similar population with the same dose of fish and soy oil showed an important increase of EPA (eicosopentanoic acid) and DHA (docosahexanoic acid) in the fish oil group after 5 months of supplementation (394% and 140%, respectively), and a moderate increase in EPA (87%) and no significant changes in DHA in the soy oil group; EPA and DHA were significantly higher in the fish oil group (*p* < 0.01) (Romieu et al. 2005) [Supplemental Material, Table 1 (online at http://www.ehponline.org/members/2008/10578/suppl.pdf)].

### Biomarkers of responses to oxidative stimuli

Characteristics of subjects who provided blood samples in the two phases of the study (presupplementation and supplementation phases) (*n* = 45) and those who did not (*n* = 7) were similar. At baseline (presupplementation phase), Cu/Zn SOD activity, LPO products, and GSH levels in plasma were similar in both supplementation groups (Table 1).

We first modeled the data from participants in both supplementation groups and evaluated the impact of supplementation and exposure to PM _2.5_ on these biomarkers (Table 2). Supplementation significantly increased the Cu/Zn SOD activity (β = 0.27, *p* = 0.002), significantly decreased levels of LPO products (β = −4.52, *p* = 0.03), and significantly increased levels of GSH in plasma (β = 0.95, *p* < 0.001). Indoor PM_2.5_ levels were inversely related to Cu/Zn SOD activity (β = −0.05, *p* = 0.001). The relation between GSH and PM_2.5_ levels was nonlinear (significant quadratic term for PM_2.5_ levels) (Table 2).

We further stratified data by supplementation group (Table 3). As observed in the overall analysis of both supplementation groups, supplementation appeared to have a protective effect in the fish oil group (significant positive association with Cu/Zn SOD activity, β = 0.29, *p* = 0.04; inverse association with LPO products, β = −3.72, *p* = 0.002; positive association with GSH levels, β = 1.0, *p* < 0.001); in the soy oil group, supplementation was positively related to Cu/Zn SOD activity (β = 0.17, *p* = 0.05) and GSH levels (β = 0.70, *p* = 0.003). We observed no effect of soy oil supplementation on LPO products (Table 3). Based on models presented in Table 3, we calculated that, compared with baseline, supplementation with fish oil led to an approximate increase in Cu/Zn SOD activity of 49.1% [95% confidence interval (CI), 2.4–81.5] and in GSH levels of 62% (95% CI, 43.3–89.0%), and an approximate decrease in LPO products of 72.5% (95% CI, 15–172%). For the soy oil group, we calculated an increase of 22.6% (95% CI, 0.7–44.0%) in Cu/Zn SOD activity and of 55.3% (95% CI, 36.3–85.1%) in GSH levels compared with baseline.

PM_2.5_ levels were inversely related to Cu/Zn SOD activity in the soy oil group (β = −0.06, *p* < 0.001) (Table 3). In the fish oil group, the relations between these biomarkers of response to oxidative stimuli and PM_2.5_ levels were nonlinear. Both a linear term and a quadratic term for PM_2.5_ levels were significant predictors of these biomarkers (Table 3).

**Figure 2:**
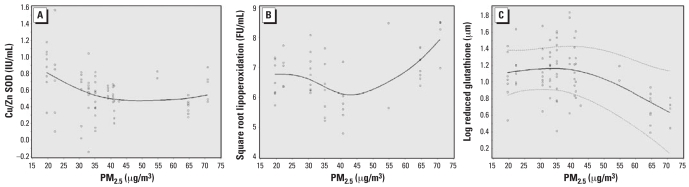
Cu/Zn SOD (*A*), LPO (*B*), and GSH (*C*) according to PM_2.5_ exposure (μg/m^3^; based on our GAMMs) among patients in the fish oil group. Solid line, estimated smooth function; dashed lines, pointwise 95% CIs; circles, partial residuals. We suppressed CIs in cases where we estimated one smooth function to be linear or any variance component to be (almost) zero, because the covariance matrix is then singular and cannot be inverted for construction of the variability bands.

**Figure 3:**
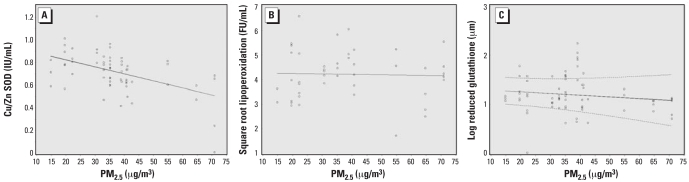
Cu/Zn SOD (*A*), LPO (*B*), and GSH (*C*) according to PM_2.5_ exposure (μg/m^3^; based on our GAMMs) among patients in the soy oil group. Solid line, estimated smooth function; dashed lines, pointwise 95% CIs; circles, partial residuals. We suppressed CIs in cases where we estimated one smooth function to be linear or any variance component to be (almost) zero, because the covariance matrix is then singular and cannot be inverted for construction of the variability bands.

To better evaluate the shape of the association between PM_2.5_ levels and the biomarkers of interest, we used GAM models. In the soy oil group, we observed that Cu/Zn SOD activity decreased linearly with increased levels of PM_2.5_. We observed no significant effect on LPO products and GSH levels (Figure 2A#x02013;C); in the fish oil group, we observed that Cu/Zn SOD activity tended to decrease with exposure to PM_2.5_ until levels came close to 40–45 μg/m^3^, and then the effect leveled off (Figure 3A). LPO products tended to decrease at low levels of PM_2.5_ and start increasing after PM_2.5_ levels of 40–45 μg/m^3^ (Figure 3B). For GSH levels, we observed little change until around 40 μg/m^3^ PM_2.5_, after which we observed a significant decrease (Figure 3C). However, because almost 75% of our PM_2.5_ measurements were < 45 μg/m^3^, our estimates at higher PM_2.5_ levels derive from sparse data.

## Discussion

In this randomized controlled trial, we observed that supplementation with both soy oil and fish oil appeared to modulate the plasma levels of biomarkers of response to oxidative stimuli by increasing Cu/Zn SOD activity and GSH levels. In addition, fish oil supplementation reduced LPO products in plasma. Fish oil appeared to modulate the adverse effect of PM_2.5_ in a nonlinear manner.

This is the first study to evaluate the impact of supplementation with n-3 PUFA on biomarkers of response to oxidative stimuli related to air pollution exposure among individuals in a noncontrolled environment. The mechanisms of PM-induced health effects are believed to involve inflammation and oxidative stress. The oxidative stress mediated by PM may arise from direct generation of ROS or be related to altered function of mitochondria or NADPH-oxidase (nicotinamide adenine dinucleotide phosphate-oxidase) and activation of inflammatory cells capable of generating ROS, reactive nitrogen species, and oxidative DNA damage (Gonzalez-Flecha 2004; Risom et al. 2005). *In vitro* studies have shown that PM exposure increases the expression of NF-κB–related genes and activation of oxidant-dependent NF-κB factors, such as tumor necrosis factor-α, and inter-leukin-8 and -6 (Gonzalez-Flecha 2004; Jimenez et al. 2000; Risom et al. 2005; Shukla et al. 2000). In animal experiments, airborne PM_2.5_ have been shown to increase levels of LPO and alter intracellular redox status in multiple organs with decreased SOD, catalase, and GSH-Px activities and depleted GSH levels (Liu and Meng 2005). Transgenic mice with overexpression of extracellular SOD (EC-SOD) had lower concentrations of oxidized glutathione in the lung after exposure to residual oily fly ash (ROFA), suggesting that enhanced EC-SOD expression decreased both lung inflammation and damage after exposure to ROFA (Ghio et al. 2002). A recent enzyme activity assay showed that the activity of Cu/Zn SOD is reduced by PM, particularly ROFA and urban PM (Hatzis et al. 2006). The impact of PM_2.5_ observed in our study is in line with those results. This impairment of defense against oxidative stress could be responsible for the decrease in HRV observed in the elderly because alteration of autonomic function related to PM_2.5_ exposure appears to be partly associated with oxidative stress (Brook et al. 2003; Chahine et al. 2007).

The major source of PM_2.5_ in Mexico City is related to vehicular traffic, and a large proportion of vehicles use diesel fuel (Secretaría de Medio Ambiente y Recursos Naturales 2003; Secretaría de Transporte y Vialidad 2006). Because smoking was not allowed in the nursing home, the main source of PM_2.5_ measured indoors came from outdoor sources, with a correlation of *r* = 0.95 between indoor and outdoor PM_2.5_ levels.

Supplementation by both fish oil containing EPA and DHA (83.2%/g) and soy oil containing ALA (6.7%), a plant-derived n-3 PUFA, appears to enhance the response to oxidative stress by increasing Cu/Zn SOD activity and GSH. The antioxidant enzyme Cu/Zn SOD appears to play an important role in response to oxidative stress, catalyzing the formation of hydrogen peroxide from superoxide anion (Rahman and Adcock 2006). GSH is a major intracellular and extracellular redox buffer and acts as a direct free radical scavenger. In animal experiments, supplementation with n-3 PUFA has been shown to have a protective effect against the toxicity of formaldehyde (FA) on the kidney. Rats administered n-3 PUFA while exposed to FA showed increased SOD and GSH-Px enzyme activities and decreased levels of malondialdehyde, a marker of LPO (Zararsiz et al. 2006). A recent study reported a roughly linear relation between DHA in human fibroblast culture and a large increase in intracellular GSH content contributing to decrease in ROS levels (Arab et al. 2006). Although in our study we measured these bio-markers in plasma, which may not parallel intracellular levels, their increase after supplementation reflects greater protection against oxidative stress. Long-chain n-3 PUFAs has been shown to act both directly (e.g., by replacing arachidonic acid as an eicosanoid substrate and inhibiting arachidonic acid metabolism) and indirectly by altering the expression of inflammatory genes through effects on transcription factor activation (Calder 2006; Maritim et al. 2003; Valko et al. 2006). The inverse association we observed with LPO products suggests also that the substitution of n-3 PUFA in the membrane could play a role in decreasing LPO of PUFA in the membranes.

Several factors need to be considered in interpreting our results. Exposure assessment was limited to a stationary 24-hr gravimetric analysis of PM_2.5_ indoors; however, the diary of daily activities kept by each participant allowed us to assess that participants spent > 93% of their time indoors and justify the use of indoor PM_2.5_ levels to determine participants’ exposure. Information about other pollutants was available through a nearby automated monitoring station, situated 3 km upwind from the study site, allowing a reasonable estimation of the nursing home air pollution atmosphere. However, we did not find an association between biomarkers of response to oxidative stimuli and other air pollutants, and these pollutants did not appear to modify the association between PM_2.5_ and biomarker levels.

Our sample size limited the detailed exploration of interactions among supplementation groups and PM_2.5_ effect. However, repeated measures in the same subject, with subjects serving as their own controls (comparing the presupplementation and supplementation phase), increased our power and accounted for slight differences in subject characteristics (Schouten 1999). The fact that the sample size and the design of the study revealed statistical evidence for an effect of supplementation with either soy oil or fish oil on Cu/Zn SOD and GSH, and with fish oil on LPO, means that the power of the study was adequate to detect these effects. The lack of significant effect of soy oil supplementation on LPO could be attributable to a real lack of effect, or to the fact that the relation of PM_2.5_ with LPO was weaker than with Cu/Zn SOD and GSH, or because the potential effect of soy oil was harder to find and therefore would have required a larger sample size. In addition, neither the laboratory technician nor the participants were aware of the randomization group minimizing the likelihood of information bias.

We based compliance with the supplementation on direct observation from the medical team, because supplements were provided to the residents of the nursing home in the morning. However, in a study conducted in a similar population with a similar design, supplementation with fish oil led to a significant increase of EPA, DHA, and n-3/n-6 PUFA ratio and a decrease of arachidonic acid whereas supplementation with soy oil led to significant increase of EPA and a marginal increase of ALA, DHA, and n-3/n-6 PUFA ratio in erythrocyte membranes (Romieu et al. 2005).

A major concern with dietary supplementation is the effective dose. The fact that fish oil appears to be more effective against oxidative stress related to PM_2.5_ exposure than is soy oil suggests that the small amount of ALA—further elongated in EPA and DHA—in soy oil might be insufficient to protect against the adverse effects of PM_2.5_ exposure. We based our results on a limited sample size but suggest that essential fatty acids might play an important role in modulating the impact of PM on health, which warrants further investigation in larger populations.

## Correction

In Table 2 of the manuscript originally published online, the intercept for LPO was 34.54; it has been corrected here.

## Figures and Tables

**Table 1:** Characteristics of the study population at baseline.

	Soy oil (*n* = 26)	Fish oil (*n* = 26)	*p*-Value
Age [mean (range)]	77 (60–88)	76 (60–96)	0.491
Male sex [no. (%)]	12 (46.2)	12 (46.2)	1.000
Smokers [no. (%)]	3 (11.5)	3 (11.5)	1.000
Medical conditions [no. (%)]			
Diabetes	2 (7.7)	6 (23.1)	0.124
Hypertension	8 (34.6)	10 (38.5)	0.773
Ischemic cardiac disease	0 (0.0)	4 (15.4)	0.037
COPD	2 (7.7)	3 (11.5)	0.638
Medical treatment [no. (%)]			
β-Blockers	1 (3.9)	2 (7.7)	0.552
Angiotensin-converting enzyme inhibitors	2 (7.7)	3 (11.5)	0.638
Calcium-channel blockers	3 (11.5)	2 (7.7)	0.638
Diuretics	0 (0.0)	3 (11.5)	0.298
BMI [mean (range)]	27.6 (20.1–37.2)	24.6 (16.6–35.9)	0.023
Oxidative stress biomarkers^a^ (mean ± SD)			
Cu/Zn SOD (IU/mL)	0.76 ± 0.04	0.68 ± 0.05	0.22
LPO (FU/mL)	18.62 ± 9.88	19.08 ± 7.90	0.85
GSH (μM)	3.66 ± 1.39	4.38 ± 1.70	0.31

a Reference values: Cu/Zn SOD, ≥ 0.75 IU/mL; LPO, 25.76–32.08 FU/mL; GSH, ≥ 5 μM.

**Table 2:** Effects of supplementation and exposure to PM_2.5_ on biomarkers of oxidative stress.

	Cu/Zn SOD		LPO^a^		GSH^b^	
	β (SE)	*p*-Value	β (SE)	*p*-Value	β (SE)	*p*-Value
Intercept	0.73 (0.04)	< 0.001	4.54 (0.52)	< 0.001	1.32 (0.08)	< 0.001
Supplementation^c^	0.27 (0.08)	0.002	−4.52 (2.00)	0.029	0.95 (0.14)	< 0.001
Fish oil^d^	−0.04 (0.04)	0.245	0.03 (0.19)	0.897	0.10 (0.09)	0.274
Indoor PM_2.5_	−0.05 (0.02)	0.001	0.08 (0.09)	0.381	0.06 (0.05)	0.176
Indoor (PM_2.5_)^2^^e^	—		—		−0.05 (0.01)	0.002

Data are regression coefficients; we adjusted models for date. For PM_2.5_, the coefficients correspond to a change in 10 μg/m^3^. The regression model for Cu/Zn SOD includes 44 subjects and 164 observations; for LPO, 42 subjects and 106 observations; for GSH, 43 subjects and 162 observations.

a Square root transformed.

b Log transformed.

c Reference category: presupplementation.

d Reference category: soy oil.

e Linear relationship; indoor (PM_2.5_)^2^ does not apply.

**Table 3:** Effects of PM_2.5_ exposure on biomarkers of oxidative stress by supplementation groups.

	Soy oil		Fish oil	
	β (SE)	*p*-Value	β (SE)	*p*-Value
Cu/Zn SOD				
Intercept	0.75 (0.03)	< 0.001	0.60 (0.06)	< 0.001
Supplementation^a^	0.17 (0.09)	0.050	0.29 (0.14)	0.042
Indoor PM_2.5_	−0.06 (0.02)	< 0.001	−0.17 (0.05)	0.002
Indoor (PM_2.5_)^2^^b^	—		0.04 (0.02)	0.009
LPO^c^				
Intercept	4.15 (0.23)	< 0.001	3.96 (0.21)	< 0.001
Supplementation^a^	0.49 (1.13)	0.669	−3.72 (1.04)	0.002
Indoor PM_2.5_	−0.02 (0.14)	0.904	−0.35 (0.19)	0.080
Indoor (PM_2.5_)^2^	—		0.16 (0.07)	0.024
GSH^d^				
Intercept	1.21 (0.10)	< 0.001	1.44 (0.08)	< 0.001
Supplementation^a^	0.70 (0.22)	0.003	1.00 (0.19)	< 0.001
Indoor PM_2.5_	−0.03 (0.04)	0.406	−0.09 (0.04)	0.017

Data are regression coefficients; we adjusted models for date. For PM_2.5_, the coefficients correspond to a change in 10 μg/m^3^. Regression models for Cu/Zn SOD include 20 subjects and 74 observations in the soy oil group and 24 subjects and 90 observations in the fish oil group; for LPO, 21 subjects and 51 observations in the soy oil group and 21 subjects and 55 observations in the fish oil group; for GSH, 20 subjects and 76 observations in the soy oil group and 23 subjects and 86 observations in the fish oil group.

a Reference category: presupplementation.

b Linear relationship; indoor (PM_2.5_)^2^ does not apply.

c Square root transformed.

d Log transformed.
